# The activation of SRC family kinases and focal adhesion kinase with the loss of the amplified, mutated *EGFR* gene contributes to the resistance to afatinib, erlotinib and osimertinib in human lung cancer cells

**DOI:** 10.18632/oncotarget.19982

**Published:** 2017-08-07

**Authors:** Yuichi Murakami, Kahori Sonoda, Hideyuki Abe, Kosuke Watari, Daiki Kusakabe, Koichi Azuma, Akihiko Kawahara, Jun Akiba, Chitose Oneyama, Jonathan A. Pachter, Kazuko Sakai, Kazuto Nishio, Michihiko Kuwano, Mayumi Ono

**Affiliations:** ^1^ Department of Pharmaceutical Oncology, Graduate School of Pharmaceutical Sciences, Kyushu University, Fukuoka, Japan; ^2^ Cancer Translational Research Center, St. Mary’s Institute of Health Sciences, Fukuoka, Japan; ^3^ Department of Diagnostic Pathology, Kurume University Hospital, Fukuoka, Japan; ^4^ Physical Chemistry for Life Science Laboratory, Faculty of Pharmaceutical Sciences, Kyushu University, Fukuoka, Japan; ^5^ Division of Respirology, Neurology and Rheumatology, Department of Internal Medicine, Kurume University School of Medicine, Fukuoka, Japan; ^6^ Division of Microbiology and Oncology, Aichi Cancer Center Research Institute, Nagoya, Japan; ^7^ Verastem Inc., Cambridge, MA, USA; ^8^ Department of Genome Biology, Kinki University Faculty of Medicine, Osaka, Japan

**Keywords:** afatinib resistance, SRC family kinase, focal adhesion kinase, non-small cell lung cancer

## Abstract

Second- and third-generation inhibitors of epidermal growth factor receptor (EGFR) tyrosine kinase activity (EGFR-TKIs) are improving the treatment of patients with non-small cell lung cancer. Here we established two sublines (BR1-8 and BR2-3) resistant to a second-generation inhibitor, afatinib, from the human lung cancer cell line HCC827 that harbors a mutation that activates the tyrosine kinase activity of EGFR. These afatinib-resistant sublines were resistant to first-generation EGFR-TKIs, gefitinib and erlotinib, and a third-generation EGFR-TKI, osimertinib. These resistant sublines showed markedly reduced levels of multiple EGFR family proteins, including the activated mutant EGFR, and complete loss of EGFR amplification as compared with their parental HCC827 cells harboring amplification of *EGFR* gene. Treatment with the multikinase inhibitor dasatinib or transfection with a *SRC* small interfering RNA inhibited cell survival and AKT phosphorylation in drug-resistant sublines to a greater extent compared with HCC827 cells. Further, the migration of drug-resistant cells was greater compared with that of HCC827 cells and was inhibited by dasatinib or an FAK inhibitor. These findings indicate that compensatory activation of SRC family kinases (SFKs) and FAK supports the survival and migration of afatinib-resistant cells when the expression of multiple EGFR family proteins was mostly abrogated. Combinations of potent drugs that target SFKs and FAK may overcome the resistance of lung cancer cells to second-generation TKIs.

## INTRODUCTION

Somatic mutations in the epidermal growth factor receptor (*EGFR*) gene that activate EGFR tyrosine kinase activity are major determinants of the clinical efficacy of first-generation EGFR tyrosine kinase inhibitors (TKIs) such as gefitinib and erlotinib that are used to treat patients with non-small cell lung cancer (NSCLC) [1-5]. Treatment with EGFR-TKIs benefits most patients with NSCLC with activating EGFR mutations, although their final clinical efficacy varies, because tumors develop resistance [6]. The acquired resistance to EGFR-TKIs is mediated through pleiotropic changes of *EGFR* gene and bypass signaling molecules [6-15]. The EGFR T790M mutant is most often responsible for mediating resistance to gefitinib and erlotinib [15].

Multikinase-targeted irreversible second-generation EGFR-TKIs such as afatinib that targets EGFR T790M have been further developed to overcome resistance to EGFR-TKIs of patients with relapsed NSCLC [6, 16-18]. Further, targeting EGFR and its family members using a combination of afatinib and cetuximab achieved improved therapeutic efficacies against acquired drug-resistant lung cancers with or without the EGFR T790M mutation [19]. Moreover, EGFR T790M-mediated drug resistance is overcome, even partially, using afatinib or other second-generation TKIs alone in preclinical models [15, 20]. The irreversible third-generation EGFR-TKI osimertinib that targets EGFR T790M shows promising responses against an activated mutant EGFR with a T790M mutation in a tumor xenograft model as well as in a clinical trial [21]. The therapeutic efficacy of osimertinib is therefore expected to provide benefits against EGFR T790M-driven acquired drug-resistant tumors [6]. For example, osimertinib is highly active in patients with lung cancer with the EGFR T790M mutation who experience disease progression during prior therapy using EGFR-TKIs [22].

Second- and third-generation receptor TKIs in combination or alone show promise for improving therapeutics against lung tumors that are refractory to erlotinib and gefitinib [22, 23]. However, the appearance of tumors resistant to EGFR T790M-targeted drugs such as osimertinib, WZ4002, and rociletinib has continuously caused serious problems for treating patients with lung cancer [6]. Moreover, further introduction of novel mutations including C797S in the TK domains of EGFR, in addition to T790M and activating mutations such as L858R or exon19 deletion, is closely associated with acquired resistance to third-generation receptor TKIs, including osimertinib [24-26]. Further, acquired resistance to osimertinib is associated with RAS signaling in lung cancer cells harboring activating EGFR mutations with EGFR T790M [27] as well as the appearance of cancer cells harboring EGFR T790M with wild-type EGFR in refractory tumors [28].

We previously established afatinib-resistant sublines from the human lung cancer cell line PC9 that harbors an activating EGFR mutation [29]. We found that expression of most EGFR family proteins in the afatinib-resistant sublines is decreased and is accompanied by activation of the FGF2/FGFR1-driven cell growth and survival signaling pathways [29]. In the present study, we further characterized afatinib-resistant sublines that were independently established from the human lung cancer cell line HCC827 harboring an activated mutant EGFR and amplification of *EGFR*. Here we report that activation of SRC family kinases SFKs and focal adhesion kinase (FAK) is responsible for the survival of afatinib-resistant cells when expression of multiple EGFR family proteins and other receptors is diminished. We discuss the potential utility of a combination of inhibitors of SFK, FAK, or both to overcome the resistance of cancer cells to multiple EGFR-TKIs.

## RESULTS

### Afatinib-resistant cells are resistant to EGFR-TKIs

We established two afatinib-resistant sublines, HCC827/BR1-8 (BR1-8) and HCC827/BR2-3 (BR2-3), after exposing the parental lung cancer cell line HCC827, which harbors a mutation of EGFR exon 19 (E746-A750) that activates tyrosine kinase activity, to step-wise increasing concentrations of afatinib up to 1 μmol/L (see Materials and Methods). We were unable to isolate drug-sensitive revertants when these resistant sublines were continuously cultured for >11 months in the absence of afatinib. BR1-8 cells grew more sparsely, with fewer cell-cell contacts and exhibited a more fibroblast-like morphology compared with HCC827 or BR2-3 cells (Supplementary Figure 1A). The doubling times of HCC827, BR1-8, and BR2-3 cells were 27 h, 22 h, and 20 h, respectively.

**Figure 1:**
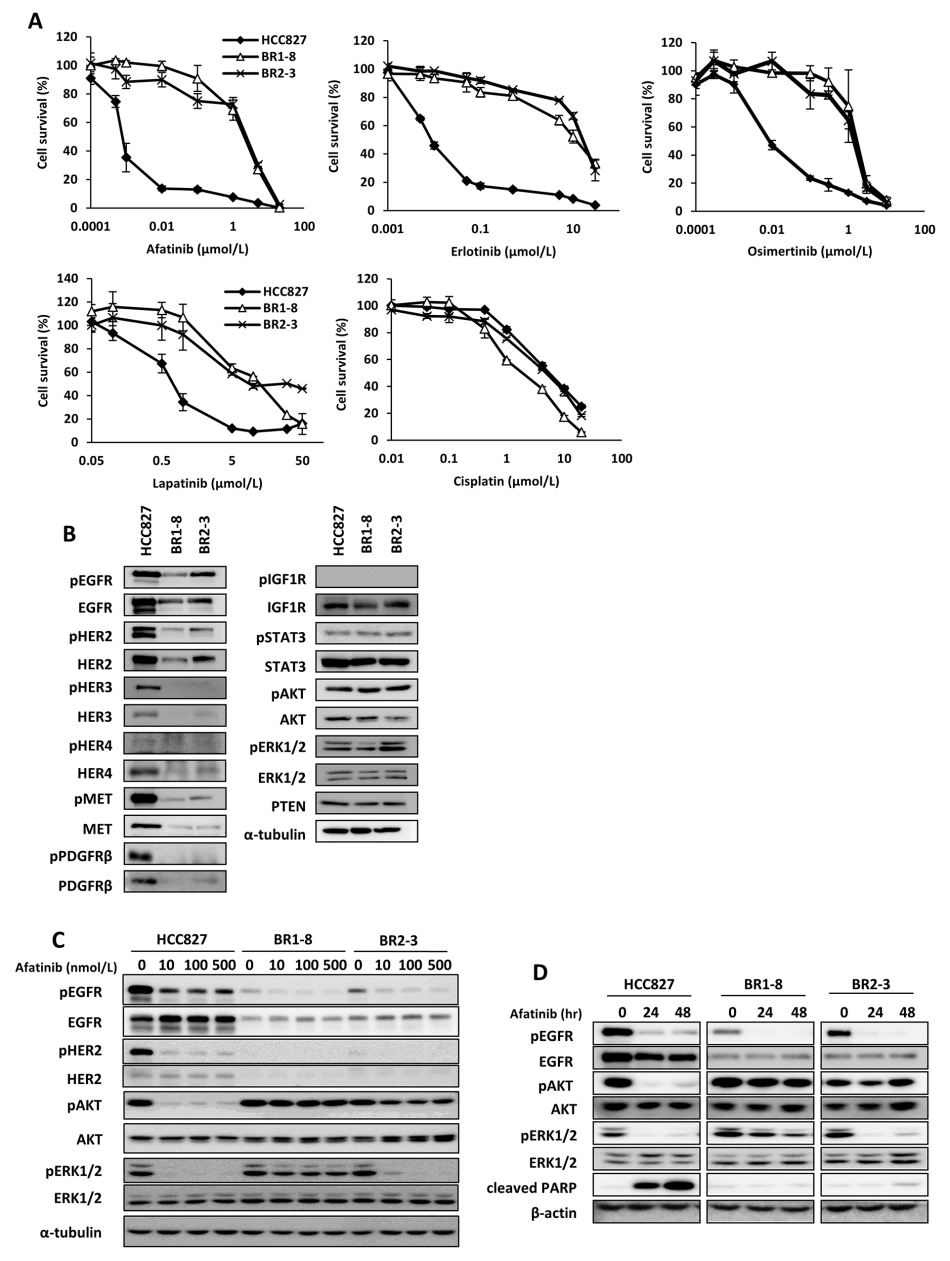
Comparison of expression and activation of receptor tyrosine kinases and the down-stream signaling molecules in HCC827 cells and its drug-resistant sublines in the absence or presence of afatinib **(A)** The sensitivities of HCC827 and drug-resistant sublines to afatinib, erlotinib, osimertinib, lapatinib, and cisplatin. Cells were exposed to various concentration of afatinib for 72 h and subjected to a WST assay. **(B)** The expression and activation of receptor tyrosine kinases and downstream signaling molecules. α-Tubulin served as the loading control. **(C)** Cells were treated for 6 h with various concentrations of afatinib. **(D)** Cells were exposed to afatinib (100 nmol/L) for 24 h or 48 h.

Compared with HCC827 cells, the BR1-8 and BR2-3 were 1,000-fold more resistant to afatinib, gefitinib, and erlotinib; approximately 3-fold more resistance to lapatinib; and >150-fold more resistant to osimertinib (Figure 1A and Table 1). In contrast, the sensitivities of the sublines to picropodophyllin, SU11274, PD173074, AZD4547, sorafenib, BIBF1120 and cisplatin were similar to those of HCC827 cells (Figure 1A and Table 1). When HCC827 cells were exposed to afatinib at 100 nmol/L for 24 h or 48 h, flow cytometric analysis detected an increased population of apoptotic cells that accumulated at the sub-G1 phase of the cell cycle compared with the resistant sublines (Supplementary Figure 1B).

**Table 1:** Comparison of sensitivities of HCC827 cells and its drug-resistant sublines to tyrosine kinase inhibitors targeting downstream signaling components and nonreceptor tyrosine kinases

Drugs	Targets	Relative drug resistance		
		HCC827	BR1-8	BR2-3
Afatinib	EGFR, HER2, HER4	1	2613	3033
Gefitinib	EGFR	1	1625	1850
Erlotinib	EGFR	1	1306	1950
Osimertinib	EGFR	1	167	154
Lapatinib	EGFR, HER2	1	3.87	3.78
picropodophyllin	IGF1-R	1	1.0	1.1
SU11274	c-Met	1	1.2	1.1
PD173074	FGFR1,3	1	1.0	1.4
AZD4547	FGFR1,2,3,4	1	1.0	1.1
Sorafenib	Raf, PDGFR, VEGFR	1	1.1	0.9
BIBF1120	PDGFR, VEGFR, FGFR	1	1.2	1.8
Cisplatin	DNA	1	0.3	0.9

IC50 values (μmol/L) were calculated from the logit regression curves of triplicate dishes. The IC_50_ values for afatinib, gefitinib, erlotinib, osimertinib, lapatinib, picropodophyllin, SU11274, PD173074, AZD4547, sorafenib, BIBF1120, and cisplatin were as follows: 0.00079±0.00005, 0.0078±0.001, 0.0087±0.0004, 0.0090±0.0019, 4.33±0.82, 0.61±0.04, 4.42±0.23, 11.50±0.32, 7.08±0.30, 5.50±0.55, 1.96±0.09, and 5.55±1.34 in HCC827, 2.05±0.25, 12.60±0.716, 11.31±0.32, 1.50±0.50, 16.76±1.63, 0.61±0.04, 5.16±0.17, 12.04±2.31, 6.94±0.41, 6.06±0.21, 2.33±0.11, and 1.90±0.33 in BR1-8 cells, and 2.38±0.31, 14.33±0.813, 16.97±0.76, 1.39±0.14, 16.38±1.28, 0.68±0.03, 5.04±0.08, 16.53±0.53, 7.67±0.40, 4.99±0.12, 3.5±0.4, and 4.98±0.91 in BR2-3 cells, respectively. Relative resistance was defined as the IC50 ±S.D. values divided by the IC50 values of the parental HCC827 cells.

### The loss of multiple EGFR family proteins in afatinib-resistant cells

We next performed western blotting analysis to compare the levels of expression of growth factor receptors and their downstream signaling components in HCC827 cells and the afatinib-resistant sublines (Figure 1B). The afatinib-resistant sublines expressed markedly decreased levels of EGFR, phosphorylated (p)EGFR, HER2, pHER2, HER3, pHER3, HER4, MET, pMET, PDGFRβ, and pPDGFRβ compared with HCC827 cells. There was no detectable change in the levels of IGF1R, STAT3, pSTAT3, AKT, pAKT, ERK1/2, pERK1/2, and PTEN among the three cell lines (Figure 1B).

We determined the effects of afatinib on the phosphorylation of EGFR, HER2, AKT and ERK1/2 (Figure 1C and 1D). Phosphorylation of EGFR and HER2 was inhibited by afatinib in HCC827 cells and the afatinib-resistant sublines as a function of its concentration (Figure 1C) as well as time (Figure 1D). In contrast, AKT phosphorylation was not detectably inhibited by afatinib in both resistant sublines, although AKT phosphorylation in HCC827 cells was completely inhibited (Figure 1C and 1D). Phosphorylation of ERK1/2 was inhibited by afatinib in all three cell lines, although ERK1/2 phosphorylation was slightly less sensitive to the inhibitory effects of afatinib in BR1-8 cells compared with HCC827 or BR2-3 cells. Apoptosis was induced in HCC827 cells upon exposure to afatinib, but not in BR1-8 and BR2-3 cells (Figure 1D).

### *EGFR* is not amplified in afatinib-resistant cells

**Figure 2:**
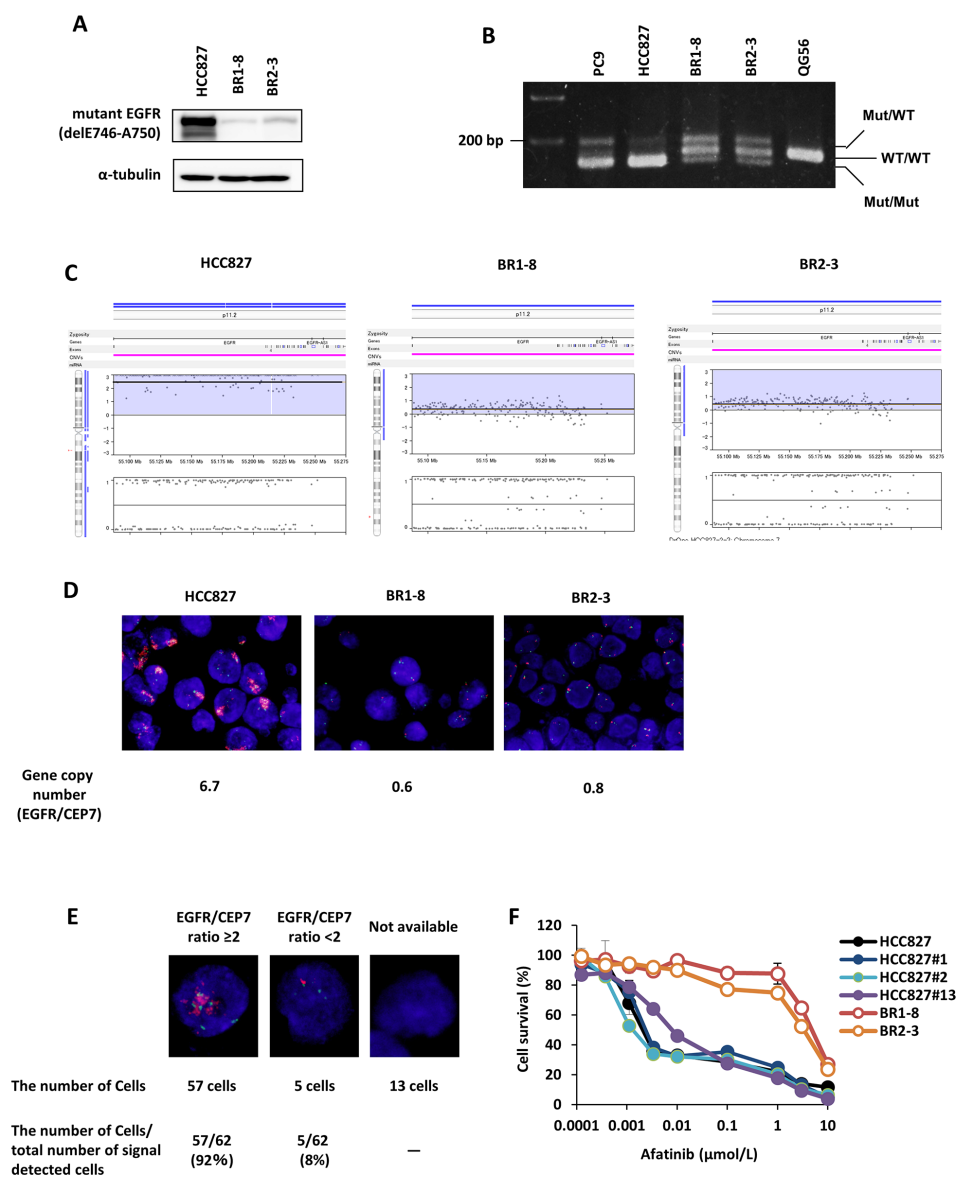
EGFR gene amplification in drug-resistant sublines **(A)** Decreased expression of delE746-A750 EGFR in drug-resistant sublines compared with HCC827 cells. **(B)** Levels of mutant and wild-type *EGFR*. PCR analysis detected only mutant homoduplexes (Mut/Mut) in HCC827 cells, and wild-type (WT/WT) homoduplexes in QG56 cells harboring wild-type EGFR, and heteroduplexes (Mut/WT) and homoduplexes (WT/WT) and (Mut/Mut) in BR1-8, BR2-3, and PC9 cells harboring the EGFR delE746-A750 mutant. **(C)** Alterations of the coding region of *EGFR* on chromosome 7 in HCC827 cells and drug-resistant sublines were determined using an Oncoscan array. The upper and lower plots show log_2_ ratios and B-allele frequencies, respectively. **(D)** FISH analysis using *EGFR* (red) and chromosome 7 centromere (CEP7) (green) probes of HCC827 cells and drug-resistant sublines. The number of the fluorescent signals corresponding to *EGFR* or CEP7 was counted, and the *EGFR*/CEP7 ratio was calculated. **(E)** The presence and absence of EGFR gene amplification of 75 cells of HCC827. FISH analysis was assessed by using EGFR (red) and chromosome 7 centromere (CEP7) (green) probes. The number of the fluorescent signals corresponding to EGFR or CEP7 was counted. **(F)** Cellular sensitivity of three clones of HCC827 (#1, #2 and #13) with or without EGFR gene amplification to afatinib. Cells were exposed to various concentrations of afatinib for 72 h and viability was assessed using a WST assay.

The loss of the gene encoding constitutively activated mutant EGFR is required for resistance to EGFR-TKIs in lung cancer cells [30]. Western blot analysis revealed markedly decreased levels of delE746-A750 EGFR in the afatinib-resistant sublines (Figure 2A). PCR analysis of genomic DNA revealed that the band specific for *EGFR* exon 19 del was less intense compared with that of the wild-type exon 19 *EGFR* sequence in the resistant sublines (Figure 2B).

*EGFR* is amplified in HCC827 cells [31]. Therefore, we analyzed *EGFR* amplification in the afatinib-resistant sublines using an Oncoscan assay and fluorescence in situ hybridization (FISH). Figure 2C shows a karyoview of the *EGFR* coding region on chromosome 7 and its copy number. *EGFR* was amplified in HCC827 cells but not in the afatinib-resistant sublines. Consistent with the results of the Oncoscan assay, FISH analysis detected *EGFR* amplification in HCC827 cells (EGFR/chromosome 7 centromere [CEP7] = 6.7) and the loss of amplification in the afatinib-resistant sublines (EGFR/CEP7 = 0.6 and 0.8 in BR1-8 and BR2-3 cells, respectively) (Figure 2D).

Concerning the mechanism by which the loss of *EGFR* gene amplification is induced during selection by resistance to afatinib, one can further ask whether afatinib-resistant cells without EGFR gene amplification are selected from the parental HCC827 cell population with and without EGFR gene amplification. We further counted cell number with or without *EGFR* gene amplification in Figure 2E. Of all 75 cells, 57 showed EGFR/CEP7 ratio >2, 5 showed EGFR/CEP7 ratio <2, and 13 showed no fluorescent signal of EGFR and CEP7 (Figure 2E). Furthermore, we independently cloned 10 cells from the parental HCC827 cells, and the absence or presence of gene amplification was analysed by FISH (Supplementary Table 1). Of the 10 clones, 9 clones showed EGFR gene amplification (EGFR/CEP7 ratio >2), but 1 clone (#13) did not show gene amplification (EGFR/CEP ration<2) (Supplementary Table 1). Dose response curves to afatinib of three clones, HCC827#1 (EGFR gene amplified), HCC827#2 (EGFR gene amplified) and HCC827#13 (EGFR gene unamplified) showed only a slight if any difference in their sensitivities to afatinib (Figure 2F). It is less likely that isolation of afatinib-resistant sublines, BR1-8 and BR2-3, is due to selection by the drug of cell population without EGFR gene amplification.

### Dasatinib inhibits cell survival and the phosphorylation of AKT and MAPK of afatinib-resistant sublines

**Figure 3:**
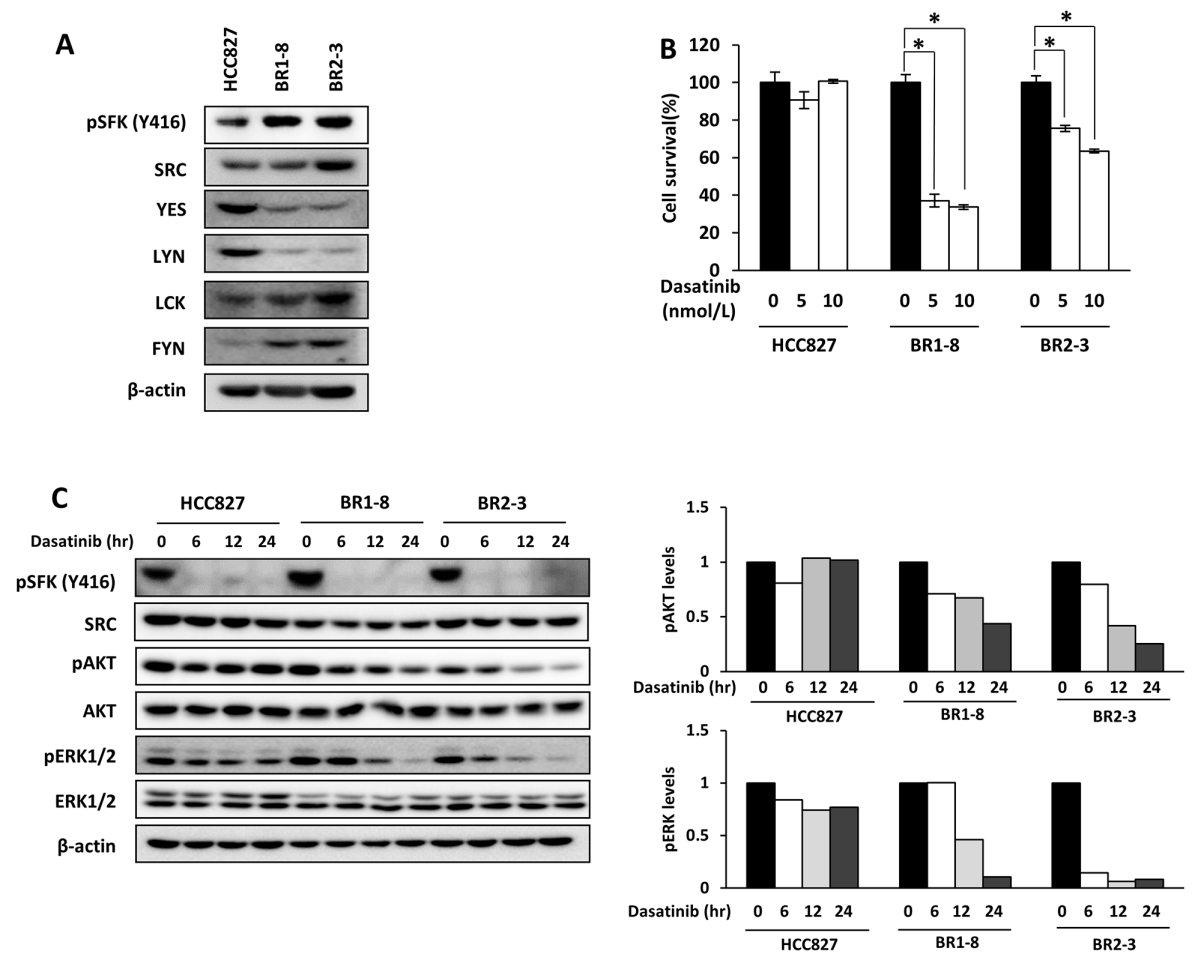
The effect of dasatinib on AKT and ERK signaling in HCC827 cells and its drug-resistant sublines **(A)** The expression of SFK family proteins and activation of SFKs. **(B)** The inhibitory effects of dasatinib on cell survival of HCC827, BR1-8, and BR2-3 cells. Each value represents the average of triplicate dishes for each cell line. *P < 0.05. **(C)** Western blot analysis of the inhibitory effects of incubating cells with dasatinib (100 nmol/L) for 6, 12, and 24 h. The quantification of the western blots is shown, and the values were normalized to those of β-actin.

Compensatory activation of SRC occurs in lung cancer cells with acquired resistance to EGFR-TKIs [32, 33]. The SFKs SRC and LCK were expressed at similar levels among the three cell lines (Figure 3A). Compared with HCC827 cells, the levels of LYN and YES were lower and those of FYN were higher in the afatinib-resistant sublines (Figure 3A). Afatinib-resistant sublines showed only a slight if any increase in pSFK levels as compared with those in HCC827 cells. We examined whether afatinib-resistant sublines showed altered sensitivity to a SFK inhibitor, dasatinib. Dasatinib inhibited the survival (Figure 3B) and the phosphorylation of AKT and ERK1/2 (Figure 3C) of the afatinib-resistant sublines to greater extents compared with HCC827 cells. The intracellular distributions of SRC, YES, LYN, LCK, and FYN in the cytosol, plasma membrane, and nucleus did not differ among the afatinib-resistant sublines and HCC827 cells (Supplementary Figure 2).

**Figure 4:**
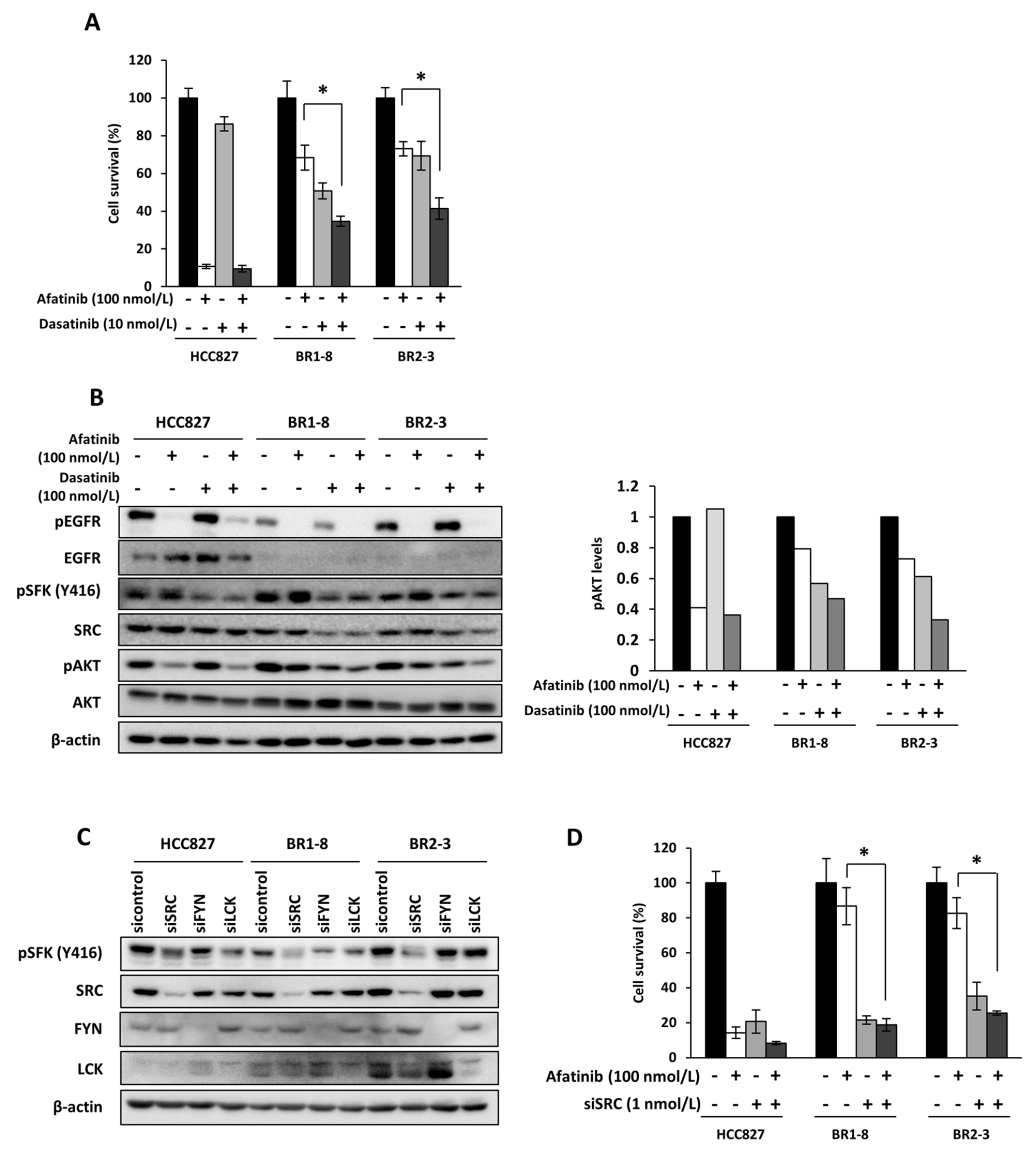
Coadministration of afatinib with dasatinib or administration of afatinib to cells transfected with a *SRC* siRNA on the survival of drug-resistant sublines **(A)** Sensitivity to dasatinib with or without afatinib. Cells were exposed for 72 h and subjected to a WST assay. *P < 0.05 vs afatinib and dasatinib treatment. **(B)** The inhibitory effect of afatinib, dasatinib, or both on AKT activation. Cells were exposed to afatinib (100 nmol/L), dasatinib (100 nmol/L), or both for 6 h. **(C)** The expression of pSFK, SRC, FYN and LCK after cells were transfected with a cognate siRNA. Cells were treated with various amounts of siRNA for 72 h. **(D)** The effect of the *SRC* siRNA on the sensitivities of cells to afatinib. Cells were transfected with the *SRC* siRNA (1 nmol/L) for 48 h and then treated with afatinib (100 nmol/L) for 72 h. Each value represents the average of triplicate dishes for each assay. *P < 0.05 vs *SRC* siRNA and afatinib treatment.

Afatinib inhibited the survival of HCC827 cell by 90% and that of the afatinib-resistant sublines by approximately 30% compared with the control (Figure 4A). Dasatinib inhibited the survival of HCC827 cells by approximately 10% and that of the afatinib-resistant sublines by 30%–50% compared with the control (Figure 4A). The combination of afatinib with dasatinib inhibited the survival of the afatinib-resistant sublines by approximately 60% (Figure 4A). Dasatinib did not inhibit AKT phosphorylation in HCC827 cells and, in contrast, inhibited AKT phosphorylation by approximately 30%–50% compared with the control in afatinib-resistant sublines (Figure 4B). SFK phosphorylation (Y416) was similarly inhibited by dasatinib in the three cell lines (Figure 4B). Coadministration of dasatinib and afatinib further inhibited AKT phosphorylation in the afatinib-resistant sublines compared with each drug alone (Figure 4B).

Knockdown of *SRC*, *FYN* and *LCK* by their cognate small interfering RNAs (siRNAs) inhibited the expression of SRC, FYN and LCK, respectively (Figure 4C). Of the three SFKs, only SRC knockdown inhibited SFK phosphorylation (Y416) in all three cell lines. However, SRC knockdown more specifically inhibited SFK phosphorylation in the resistant sublines than HCC827 (Figure 4C). *SRC* siRNA markedly and similarly inhibited the survival of the three cell lines, and the combination of afatinib and the *SRC* siRNA markedly inhibited the survival of the afatinib-resistant sublines compared with afatinib alone (Figure 4D).

### Increased expression of FGFR1, EPHA4, and EPHA2 is not directly involved in afatinib-resistance

We next determined whether other receptors could induce SRC activation in the drug-resistant sublines. We reported that in afatinib-resistant sublines derived from the lung cancer cell line PC9 that harbors an activating EGFR mutation, the levels of multiple EGFR family proteins are markedly reduced and accompanied by compensatory activation of an FGF2/FGFR1 autocrine signaling pathway [29]. Although the drug-resistant sublines expressed higher levels of FGFR1 compared with HCC827 cells, they did not express increased levels of pFGFR (Supplementary Figure 3). Further, the sensitivities of HCC827 and the drug-resistant sublines to FGFR-TKIs, PD173074 and AZD4547, were similar (Supplementary Figure 3), suggesting that FGFR1 did not act as a driver oncogene in the afatinib-resistant sublines.

Dasatinib inhibits the activities of SFKs, EPH family members, ABL, PDGFR, and KIT [34]. Dasatinib targets EPHA4 [35, 36], and here we found that EPHA4 expression was markedly increased in the drug-resistant sublines compared with HCC827 cells (Supplementary Figure 4A). Transfection with an *EPHA4* siRNA did not inhibit the phosphorylation of AKT and ERK1/2 in BR1-8 and BR2-3 cells. EPHA4 knockdown inhibited the survival of HCC827 and BR2-3 cells by approximately 20% compared with the untreated control (Supplementary Figure 4B), and knockdown of EPHA4 expression did not inhibit the survival of BR1-8 cells. On the other hand, phosphorylation of EPHA2 promotes SRC activation [37] and is involved in the acquisition of resistance of lung cancer cells to EGFR-TKIs [38]. Compared with HCC827 cells, the levels of EPHA2 were higher in BR1-8 cells and similar in BR2-3 cells (Supplementary Figure 4C). An *EPHA2* siRNA inhibited cell survival by approximately 20%–40% in the three cell lines compared with the control (Supplementary Figure 4D), and when combined with afatinib, the *EPHA2* siRNA did not affect the survival of resistant cells compared with afatinib alone (Supplementary Figure 4D).

### Increased FAK activation in afatinib-resistant sublines that migrate more extensively compared with the parental cells

**Figure 5:**
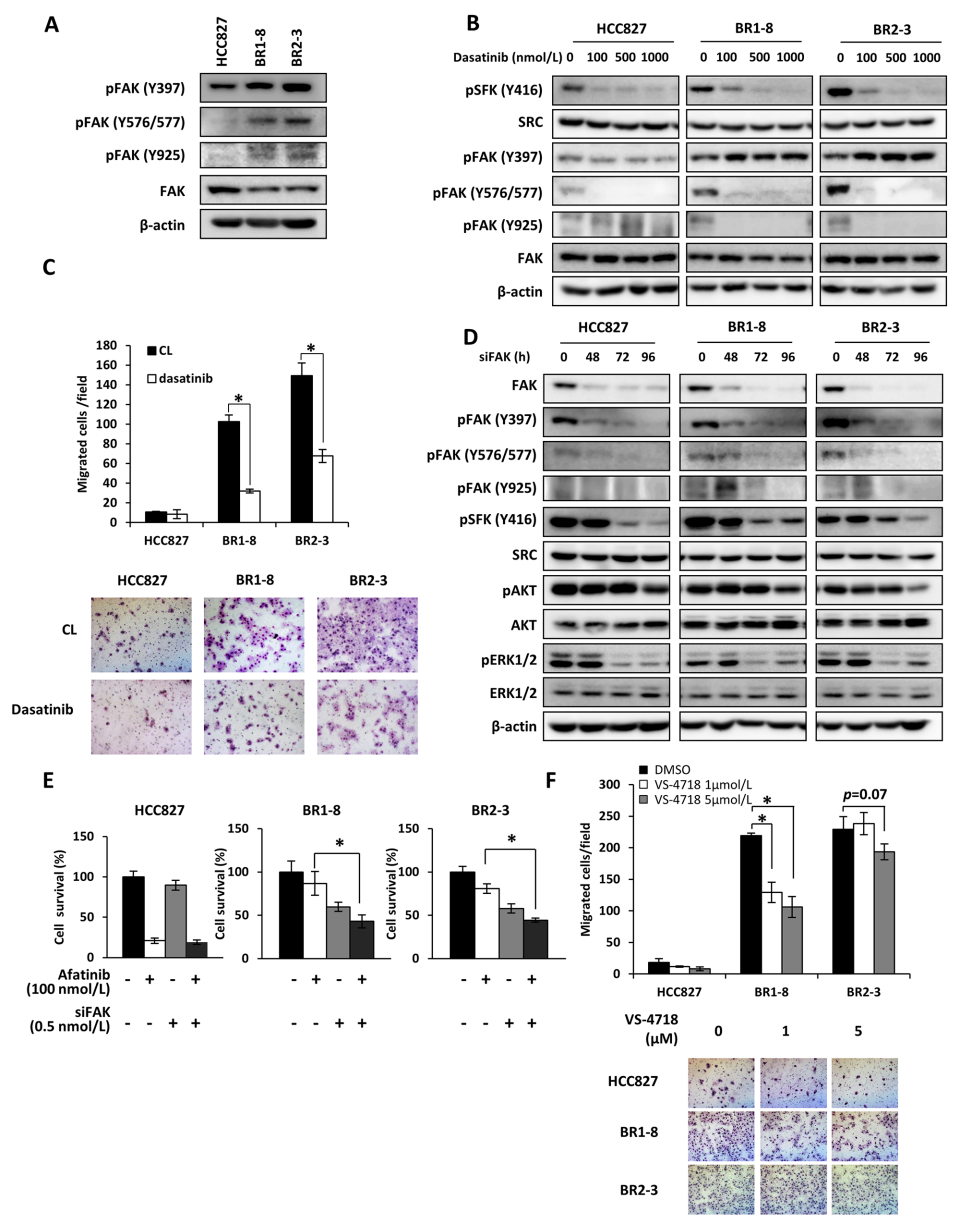
FAK activation in association with SRC in drug-resistant sublines **(A)** The expression and activation of FAK. β-Actin served as a loading control. **(B)** The inhibitory effects of dasatinib on FAK activation. Cells were exposed to dasatinib for 6 h. **(C)** Migration of HCC827 cells and drug-resistant sublines. Migration was determined using transwell assays in the presence or absence of without dasatinib for 6 h. Each value represents the average of triplicate dishes for each assay. *P < 0.05 vs dasatinib treatment. **(D)** Effects of a *FAK* siRNA on activation of downstream signaling molecules. Cells were treated with a *FAK* siRNA (0.5 nmol/L) for various times. **(E)** Effects of a *FAK* siRNA on sensitivity to afatinib. Cells were exposed to *FAK* siRNA (0.5 nmol/L) for 48 h and then treated with afatinib (100 nmol/L) for 72 h. *P < 0.05 vs *FAK* siRNA and afatinib treatment. **(F)** Inhibitory effects of VS-4718 on cell migration. Migration was determined using a transwell assay in the presence or absence of VS-4718 for 6 h. *P < 0.05 vs DMSO treatment.

FAK is closely associated with SFKs [39], and the SFK/FAK signaling pathway controls cell motility, invasion, survival, and drug resistance [32, 39, 40]. Phosphorylation of FAK (Y397, Y576/577, and Y925) in BR1-8 and BR2-3 cells was increased compared with that detected in HCC827 cells (Figure 5A). Of these phosphorylation sites, phosphorylation of Y925 was not apparently seen in HCC827 (Figure 5A, 5B, and 5D). Dasatinib inhibited phosphorylation of SFK phosphorylation (Y416), resulting in suppression of FAK (Y576/Y577) and FAK (Y925) phosphorylation in BR1-8 and BR2-3 cells (Figure 5B). Phosphorylation of FAK (Y576/Y577) was inhibited by dasatinib not only in resistant sublines but also in HCC827 cells. Phosphorylation of FAK (Y397) was slightly decreased by dasatinib in the parental HCC827 cells, but FAK phosphorylation (Y397) was increased in both resistant sublines by dasatinib (Figure 5B). The migration of drug-resistant sublines was >10-fold higher compared with that of HCC827 cells and was significantly inhibited in the presence of dasatinib (Figure 5C). Since cell migration is often associated with activation of SFK and FAK [41], increased migration of resistant cell might be attributed to the activation of SFK and FAK.

Cell growth and AKT phosphorylation are suppressed by inhibition of FAK [42]. The kinetics of siRNA-mediated FAK knockdown shows that the phosphorylation of FAK (Y397 and Y576/577) was inhibited by si*FAK* in HCC827 and the drug-resistant sublines (Figure 5D). The phosphorylation of SFKs and ERK1/2 was inhibited 72 h and 96 h after si*FAK* transfection of the three cell lines and was followed by inhibition of AKT phosphorylation at 96 h. AKT phosphorylation was inhibited to a greater extent in si*FAK*-transfected BR1-8 and BR2-3 cells compared with HCC827 cells (Figure 5D).

We next determined whether si*FAK* or a FAK inhibitor inhibited the survival and migration of the afatinib-resistant sublines. The survival of drug-resistant sublines transfected with si*FAK* was reduced by 40%–50% and that of HCC827 cells by 10% compared with the controls (Figure 5E). The survival of the drug-resistant cells treated with afatinib and simultaneously transfected with si*FAK* was inhibited by >50% (Figure 5E).

FAK activation promotes cell migration and invasion [41]. Therefore, we determined whether a FAK inhibitor inhibited the migration of the drug-resistant sublines. VS-4718 was shown to inhibit the FAK activity of cancer cells [43]. The sensitivities of afatinib-resistant sublines to VS-4718 were similar to those of HCC827 cells (data not shown). The migration of the drug-resistant sublines was 10-fold higher compared with that of HCC827 cells. VS-4718 significantly (p<0.05) inhibited the migration of BR1-8 cells and also that of BR2-3 cells (p=0.07) (Figure 5F). Together, the data indicate that activation of the SFK/FAK pathway is the main mechanism of survival and migration of afatinib-resistant cells.

## DISCUSSION

**Figure 6:**
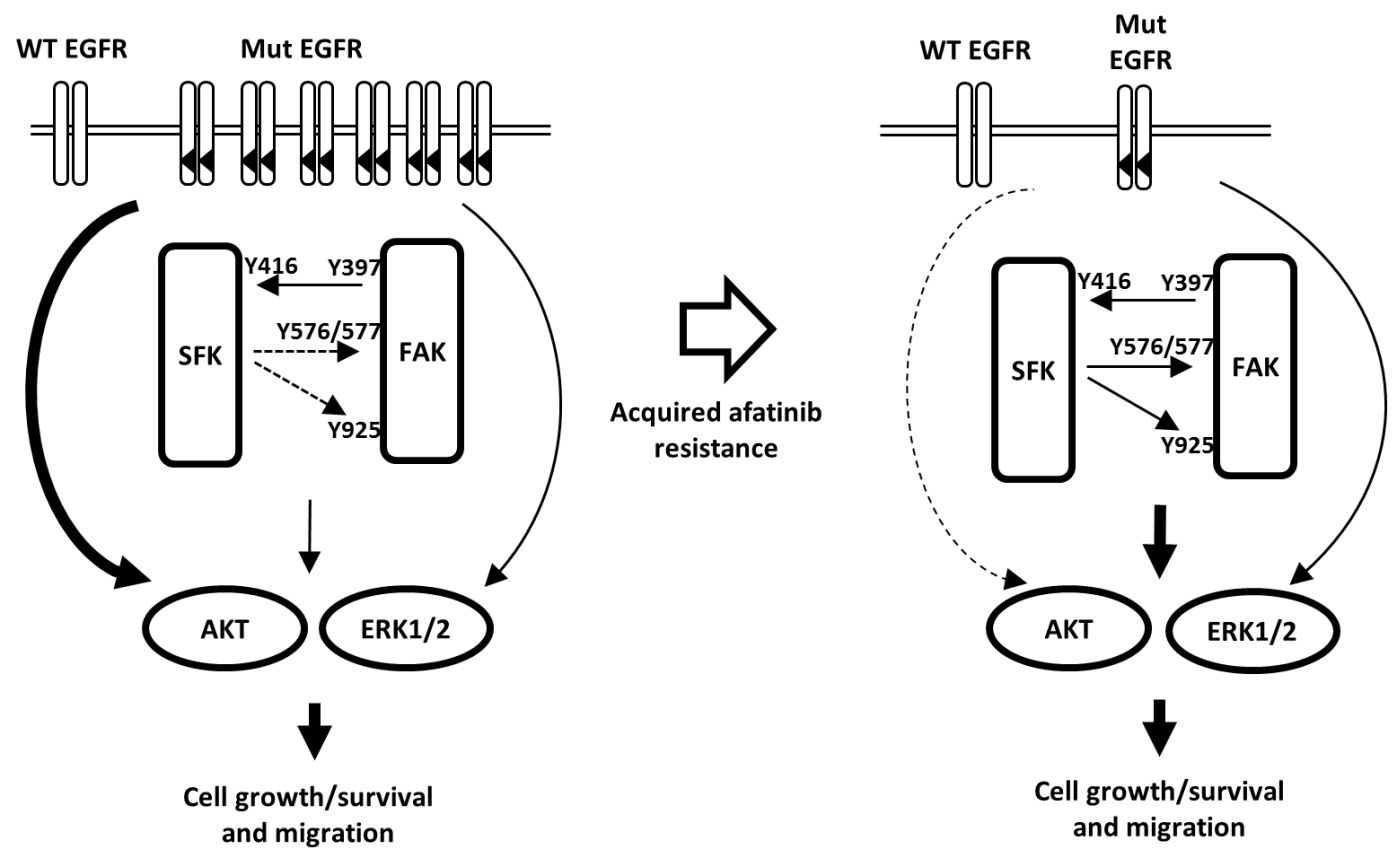
Hypothetical model illustrating the mechanism of acquisition of resistance to afatinib and the role of SFK/FAK signaling in mediating cell growth and survival Cell growth and survival of the parental drug-sensitive cell harboring activated mutant EGFR depends upon an amplified activated EGFR-driven PI3K/AKT pathway that is highly susceptible to afatinib. In contrast, in afatinib drug-resistant cells, expression of the activated mutant EGFR is markedly reduced because of the loss of EGFR gene amplification. Conversely, SFK/FAK activation contributes to growth, survival, and migration of afatinib-resistant cells.

HCC827 cells harboring an activating EGFR mutation are highly susceptible to EGFR-TKIs, and the afatinib-resistant sublines derived here from HCC827 cells showed the characteristics as follows: (1) The levels of EGFR, an activated mutant EGFR, HER2, HER3, HER4, MET, and PDGFRβ were reduced compared with those of the parental cells, and *EGFR* amplification was lost. (2) AKT phosphorylation was inhibited by the SFK inhibitor dasatinib but not by afatinib, and administration of afatinib together with dasatinib or to SRC-knockdown cells further inhibited AKT phosphorylation and cell survival. (3) FAK and SFKs were more highly activated and cell survival and migration as well as AKT phosphorylation were inhibited by FAK knockdown. On the other hand, a clinical study has reported that the EGFR-T790M mutation is present in patients with lung tumors refractory to treatment with afatinib [44]. However, we did not detect the EGFR-T790M mutation in BR1-8 and BR2-3 (unpublished data), suggesting that the T790M mutation does not contribute to the afatinib-resistance of BR1-8 and BR2-3 cells. Together, these data indicate that acquisition of afatinib resistance was likely caused by the loss of amplification of activated mutant EGFR. The survival and migration of the afatinib-resistant cells was likely mediated by the activities SFK/FAK-driven AKT and ERK signaling pathways (Figure 6). Concerning how SFK/FAK is activated by afatinib-resistance, we could not detect any mutation in *SFK* and *FAK* as well as *Eph* family, *ABL*, *PDGFR* and *KIT* (unpublish data).

Afatinib-resistant H1975 cells harboring an activated mutant EGFR and the T790M mutation express increased levels of KIT and MET in a mouse xenograft model, and combined knockdown of HER3, KIT, and MET causes cell death [45]. The combination of afatinib with dasatinib or amuvatinib, a KIT and MET inhibitor, overcomes afatinib-resistance, suggesting that compensatory activation of HER3 and SRC are likely involved in the acquired drug-resistance of tumors [45]. We previously reported that both the loss of activated mutant EGFR and the compensatory activation to other EGFR family proteins, HER2 and HER3, are involved in acquired EGFR-TKI resistance [30]. The present study shows that inhibition of SRC by dasatinib or a *SRC* siRNA inhibited the survival of the afatinib-resistant sublines when the expression of multiple EGFR family proteins, MET, and PDGFRβ is markedly reduced. Afatinib-resistant sublines thus attenuates the cell growth and survival signaling pathways driven by the activated mutant EGFR as well as by HER2, HER3 and MET, and their cell growth and survival newly depend on compensatory activation of SFK/FAK signaling pathway (See Figure 6).

We found previously that integrinβ1-driven SRC activation is involved in enhanced cell migration and invasiveness as well as the acquisition erlotinib-resistance in human lung cancer cells harboring activated mutant EGFR, and integrinβ1 knockdown or inactivation overcomes drug resistance [33]. The afatinib-resistant sublines studied here, however, did not express activated integrinβ1, and integrinβ1 knockdown did not affect SFK phosphorylation or cell survival (unpublished data), suggesting that it is less likely that the integrinβ1/SRC/AKT axis is involved in the acquisition of afatinib resistance.

SRC is a nonreceptor TK that functions as a cotransducer of signals generated by transmembrane growth factor receptors such as EGFR, and SRC plays a critical role in EGFR-induced cell growth and surviving signaling [46]. Seven of nine SRC family kinase genes are included among 18 genes that encode proteins that modify the EGFR-dependent cell growth and survival of lung cancer cells that harbor an activated mutant EGFR, [47], suggesting EGFR-independent activation of the MEK/ERK and PI3K/AKT signaling pathways [6]. EGFR-independent SRC is also activated in other EGFR-TKI-resistant lung cancer cells, and dasatinib together with EGFR-TKIs overcomes drug resistance [48]. Our present study demonstrates that afatinib-resistant cells were more susceptible to the cytotoxic effects of dasatinib or SRC knockdown. Of several SRC family genes, knockdown of SRC specifically suppressed SFK activation (pSFK Y416) in BR1-8 and BR2-3 cells, suggesting that SRC is one potent gene involved in the survival of afatinib-resistant cells (Figure 4C).

Further, knockdown of SRC inhibited the survival of afatinib resistant sublines as well as their parental HCC827 cells, and Src signaling also contributes to the survival of HCC827 cells. Dasatinib inhibited cell growth and AKT phosphorylation more strongly in afatinib resistant sublines than HCC827 cells (Figure 3B and 3C), suggesting again that the survival of afatinib resistant sublines more depend on Src signaling. However, dasatinib did not completely inhibit Akt phosphorylation in afatinib resistant sublines, suggesting that other unknown molecules in addition to SFK might be also involved in the cell survival of afatinib resistant sublines. Identification of molecule other than SFK/FAK which may contribute to cell survival of afatinib resistant sublines should be further required.

BR1-8 and BR2-3 cells showed markedly enhanced migration compared with HCC827 as well as relatively higher levels of FAK phosphorylation. Cell migration is closely associated with activation of SFK and FAK [41] and FAK functions downstream or upstream of SFKs [39]. In erlotinib-resistant cells undergoing the epithelial-mesenchymal transition, an SFK/FAK signaling pathway is key for cell survival [40]. SFKs and FAK interact in cancer cells to drive signaling pathways that mediate cell growth, survival, and migration. Further, the migration of drug-resistant cells was blocked by the FAK inhibitor VS-4718 as well as by dasatinib, suggesting that activation of SFK and FAK promotes cell migration as well as cell growth/survival by afatinib resistant sublines (Figure 6).

In conclusion, acquired resistance to the multi-EGFR family TKI afatinib was closely associated with the loss of amplification of *EGFR* harboring an activating mutation as well as with reduced levels and activities of other growth factor receptors. Further, we detected constitutive activation of SFKs/FAK that promoted the growth, survival, and migration of afatinib-resistant cells. Treatment with the SFK inhibitor dasatinib partially overcame afatinib resistance through suppression of the SFK/FAK-AKT axis and/or the SFK/FAK-ERK axis. SFK/FAK activation may therefore play a key role in growth, survival, and migration of afatinib-resistant cells.

## MATERIALS AND METHODS

### Generation and culture of afatinib-resistant sublines derived from HCC827 cells

PC9 was kindly provided by Dr. Yukito Ichinose (National Hospital Organization Kyushu Cancer Center, Fukuoka, Japan) [49]. HCC827 was purchased from the American Type Culture Collection. PC9 and HCC827 were not further tested or authenticated by the authors. These lung cancer cell lines were maintained in RPMI medium supplemented with 10% fetal bovine serum (FBS) and incubated in a humidified atmosphere containing 5% CO2 at 37°C. Afatinib-resistant sublines were established from HCC827 cells as previously described [8, 29, 30]. We cultured HCC827 cells in increasing, step-wise concentrations of afatinib up to 1 μmol/L over the following 11 months. We independently cloned two afatinib-resistant sublines from two dishes and designated them HCC827/BR1-8 and HCC827/BR2-3. The identities of these sublines were confirmed by analyzing their short tandem repeat profiles using the Cell ID System (Promega, Madison, WI). All cell lines were passaged for ≤6 months.

### Reagents

Erlotinib was kindly provided by F. Hoffman-La Roche Ltd, gefitinib was provided by AstraZeneca Inc; VS-4718 was provided by Verastem Inc; afatinib, osimertinib, lapatinib, AZD4547, and BIBF1120 were purchased from Selleck Chemicals; SU11274 and picropodophyllin were from Carbiochem; dasatinib was from Bio Vision; SB203580 was from Cayman Chemical; sorafenib was acquired from Toronto Research Chemicals Inc, cisplatin was from Bristol-Myers Squibb Company; and PD173074 was from Sigma-Aldrich.

Anti-HER2 and anti-pHER2 antibodies were purchased from Merck Millipore Corporation, anti-EGFR, anti-pEGFR, anti-pHER3, anti-HER4, anti-pHER4, anti-pc-Met, anti-IGF1Rβ, anti-pIGF1Rβ, anti-PDGFRβ, anti-pPDGFRβ, anti-FGFR1, anti-pFGFR, anti-ERK1/2, anti-pERK1/2, anti-AKT, anti-pAKT, anti-STAT3, anti-pSTAT3, anti-PTEN, anti-SRC, anti-FYN, anti-LYN, anti-YES, anti-LCK, anti-pSRC family (Y416), anti-pSRC (Y527), anti-FAK, anti-pFAK (Y397), anti-pFAK (Y576/577), anti-pFAK (Y925) and anti- EGFR (del E746-A750) antibody were from Cell Signaling Technology, anti-HER3 and anti-c-Met were from Santa Cruz Biotechnology Inc, anti-β-actin was from Abcam, Inc., and anti-α-tubulin was from Sigma-Aldrich.

### Western blot analysis

The cells were rinsed twice with ice-cold PBS and then lysed in Triton X-100 buffer (50 mmol/L HEPES, 150 mmol/L NaCl, 50 mmol/L NaF, 1% Triton X-100, and 10% glycerol containing 5 mmol/L EDTA, 1 mmol/L phenylmethylsulfonyl fluoride, 10 μg/mL aprotinin, 10 μg/mL leupeptin, and 1 mmol/L sodium orthovanadate). The proteins in the cell lysates were separated using SDS-PAGE and electrophoretically transferred to Immobilon membranes (Millipore Corp.) [30].

### WST assay

Cells were plated in 96-well flat-bottom plates and cultured for 24 h before exposure to various concentrations of drugs for 72 h at 37°C. Cell Count Reagent SF (15 μL) (Nacalai Tesque) was added to each well, and the plates were incubated for 2 to 3 h at 37°C. Absorbance was measured at 450 nm using a 96-well plate reader. Triplicate wells were tested at each drug concentration. The IC50 value was defined as the concentration that reduced absorbance by 50% and was calculated from the survival curves.

### PCR analysis

To analyze the deletion mutation, EGFR exon 19 was amplified using TaKaRa ExTaq polymerase and the PCR primers as follows: forward primer 5´-ATGTGGCACCATCTCACAATTGCC-3´, reverse primer 5´-CCACACAGCAAAGCAGAAACTCAC-3´.

### RNA interference assays

Cells were transfected with siRNA duplexes in the presence of Lipofectamine RNAiMAX and Opti-MEM medium (Thermo Fisher Scientific Inc) according to the manufacturer’s recommendations. siRNAs targeting the mRNAs encoding SRC, FAK, FYN, LCK, EPHA2, EPHA4 as well as a nonspecific (control) were purchased from Thermo Fisher Scientific Inc.

### Flow cytometry

Cells were treated with afatinib for 24 h or 48 h before flow cytometry analysis. Cells were washed twice with cold PBS, harvested, and stained with propidium iodide (Cycletest Plus DNA kit, Becton-Dickinson). Flow cytometry was conducted using a FACS Calibur System (Becton-Dickinson and Company).

### Cell migration assay

Serum-induced cell migration assays were performed using a multiwell chamber as the outer chamber with 8-μm polycarbonate filters. Cells at (2.0 × 10^6^ cells) in serum-free RPMI with or without dasatinib were seeded in the inner chamber, and 10% FBS was added to the outer chamber. After 6 h incubation, cells that migrated under the filter were counted.

### FISH analysis of EGFR copy number

Gene copy number per cell was investigated using FISH with an LSI EGFR SpectrumOrange/CEP 7 SpectrumGreen probe (Vysis Inc, Abbott Laboratories, IL) dual probe cocktail. One slide of each cytological sample was subjected to hybridization reactions. The specimen underwent pretreatment with 0.2 M HCl at room temperature for 20 min and then in sodium citrate buffer (2 × SSC, pH 6.0) at 80°C for 30 min. The specimens were digested using Proteinase K (ready to use, Dako Cytomation, Denmark) diluted 6-times with 2 × SSC at room temperature for 10 min, rinsed in sodium citrate buffer for 5 min, and dehydrated in ethanol for 2 min. After the dual-color LSI EGFR SpectrumOrange/CEP 7 SpectrumGreen probe was applied to the dry specimen, a coverslip was placed over the area containing the cells and sealed with rubber cement. The specimens were then incubated in a hybridizer (Dako Cytomation, Denmark) and subjected to denaturation at 95°C for 5 min and hybridization at 37°C for approximately 16 h. After hybridization, the samples were washed using 2 × SSC/0.3% NP-40 at 72 ± 1°C for 2 min after the addition of 10 μL of mounting medium containing 4′,6′-diamidino-2-phenylindole to the cell area that was then protected using a coverslip. The EGFR Spectrum Orange/chromosome 7 spectrum green signals were counted in a minimum of 20 cells using a fluorescence microscope (BX 51, Olympus, Japan). EGFR amplification was defined as an EGFR Spectrum Orange/chromosome 17 spectrum green signal ratio >2.0.

### Isolation of genomic DNA

Genomic DNA was extracted using the QIAamp DNA Mini Kit (Qiagen). The quality and quantity of the DNA were verified using a NanoDrop 1000 (ThermoFisher Scientific).

### Array comparative genomic hybridization

Genomic DNA was subjected to the OncoScan FFPE Assay (Affymetrix) according to the manufacturer’s protocol. DNA (80 ng) was annealed with molecular inversion probes (MIPs) for 16–18 h, followed by enzymatic digestion and a gap-fill reaction. The circular MIP probes were linearized using a restriction enzyme and amplified using PCR. PCR products were enzymatically cleaved and fragmented followed by hybridized onto the OncoScan array. After hybridization for 16–18 h, arrays were stained and washed using the GeneChip Fluidics Station 450 and loaded into a GeneChip Scanner 3000 7G (Affymetrix). Array fluorescence intensity (CEL) files were generated using the Affymetrix GeneChip Command Console software version 4.1. The CEL files were converted to OSCHP files using OncoScan Console 1.3 Software. The OSCHP file was analyzed using the TuScan algorithm and Nexus Express for Oncoscan software version 3.1 (Biodiscovery, Inc.).

### Cell fractionation assay

Cells were seeded and harvested for 3 days. Cell fractionation was performed using Subcellular Protein Fractionation Kit for Cultured Cells (Thermo Scientific Inc.) according to the manufacturer’s recommendations.

## SUPPLEMENTARY MATERIALS FIGURES AND TABLE


