# Supplementary material for: The activation of SRC family kinases and focal adhesion kinase with the loss of the amplified, mutated EGFR gene contributes to the resistance to afatinib, erlotinib and osimertinib in human lung cancer cells
Source: Oncotarget. 2017 Aug 7;8(41):70736–51. doi: 10.18632/oncotarget.19982 (PMC5642590; doi:10.18632/oncotarget.19982)
Supplement: Supplementary file 1 [file oncotarget-08-70736-s001.pdf]

# The activation of SRC family kinases and focal adhesion kinase with the loss of the amplified, mutated *EGFR* gene contributes to the resistance to afatinib, erlotinib and osimertinib in human lung cancer cells

## SUPPLEMENTARY MATERIALS

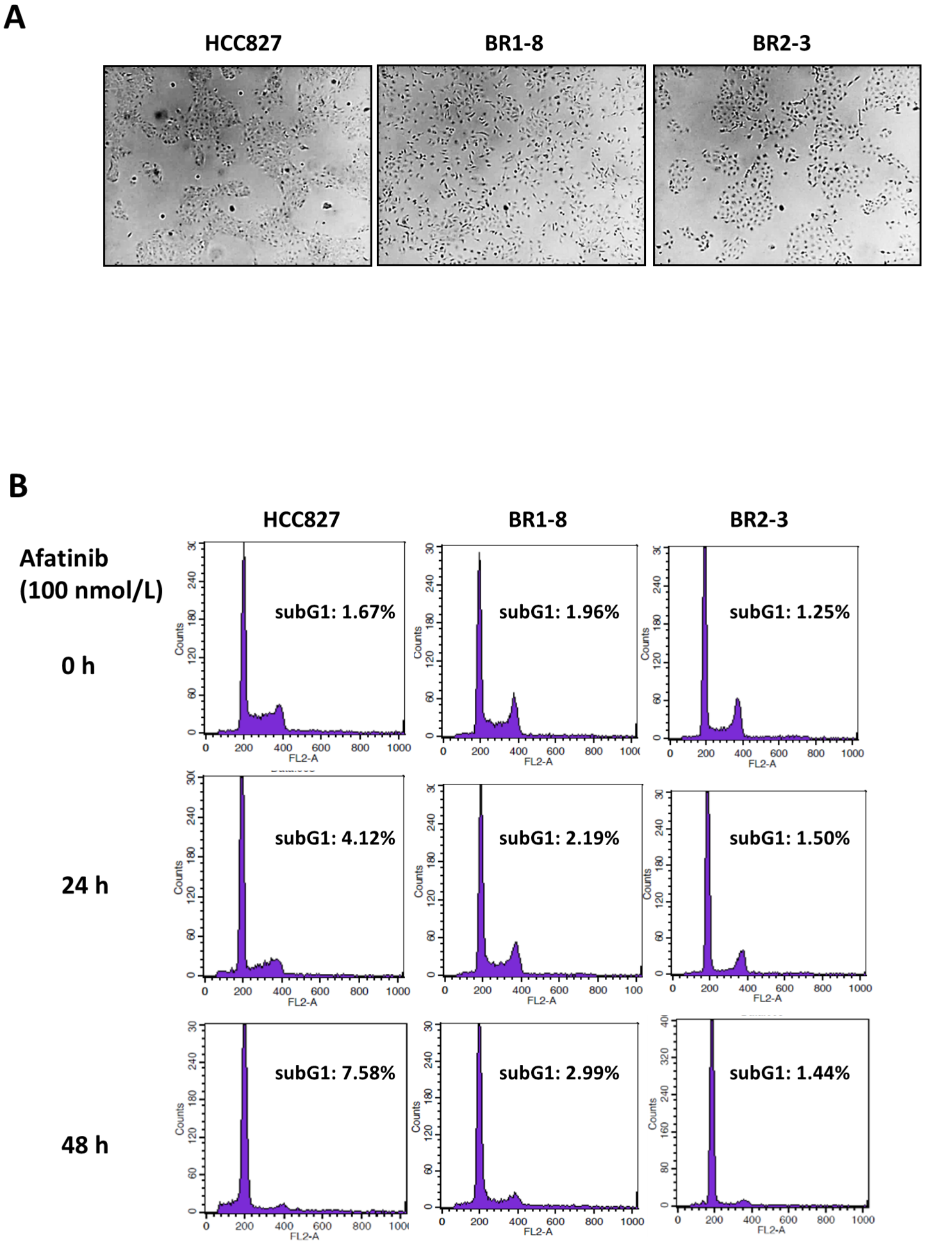


**Supplementary Figure 1: Cell morphology and increased populations of apoptotic cells in cultures of HCC827 cells and drug-resistant sublines in the presence of afatinib. (A)** Morphologies of HCC827 cells and drug-resistant sublines. **(B)** The flow cytometry profile of the cell cycle in HCC827 cells and drug-resistant sublines. Cells were treated with afatinib (100 nmol/L) for 24 h or 48 h, and the cell cycle distribution was analyzed using flow cytometry. The percentages of cells in the sub-G1 phase of the cell cycle are shown.

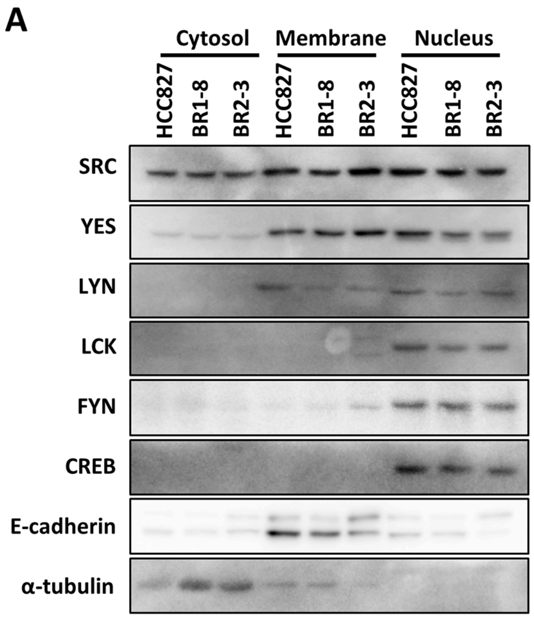


**Supplementary Figure 2: Cellular distribution of SFK proteins in HCC827, BR1-8 and BR2-3.** **(A)** Western blot analysis showed expression of SFKs in cytosol, plasma membrane and nucleus fraction, respectively. CREB, a nuclear marker; E-cadherin, a membrane marker; and α-tubulin, a cytosol marker.

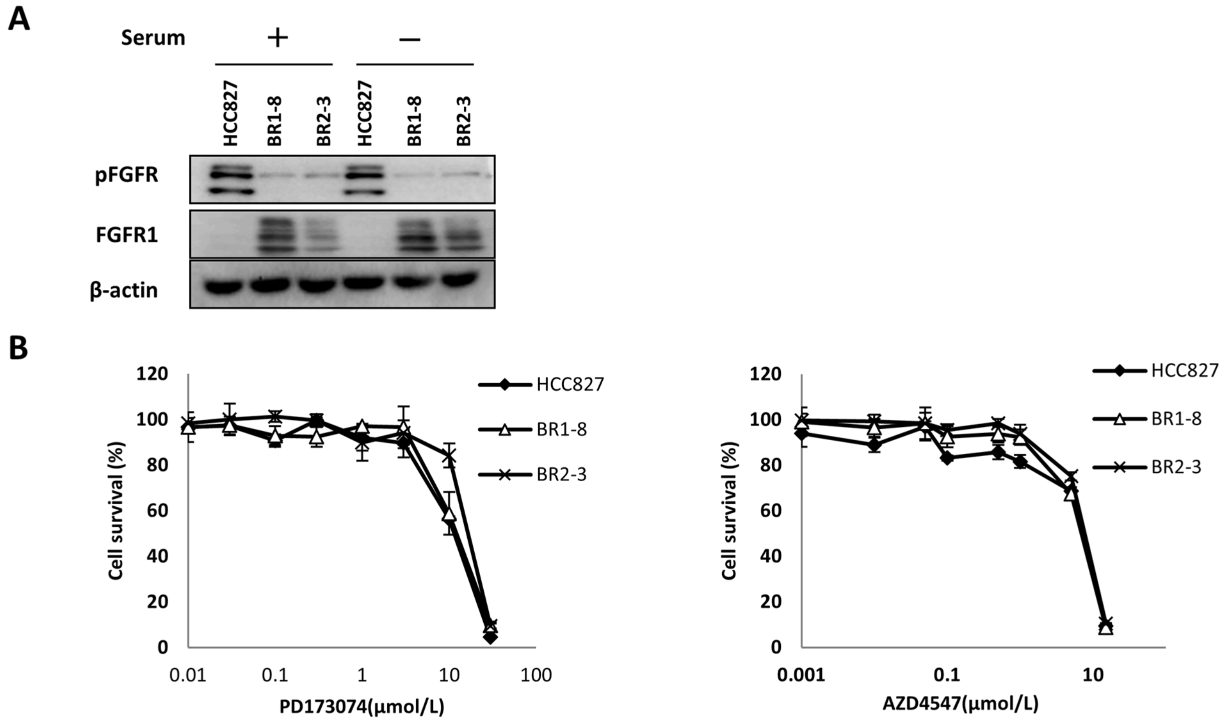


**Supplementary Figure 3: Increased expression of FGFR1 by drug-resistant sublines.** **(A)** The expression and activation of FGFR1. β-Actin served as a loading control. **(B)** The sensitivity of HCC827 cells and drug-resistant sublines to PD173074 or AZD4547. Cells were exposed to various concentrations of drugs for 72 h and viability was assessed using a WST assay.

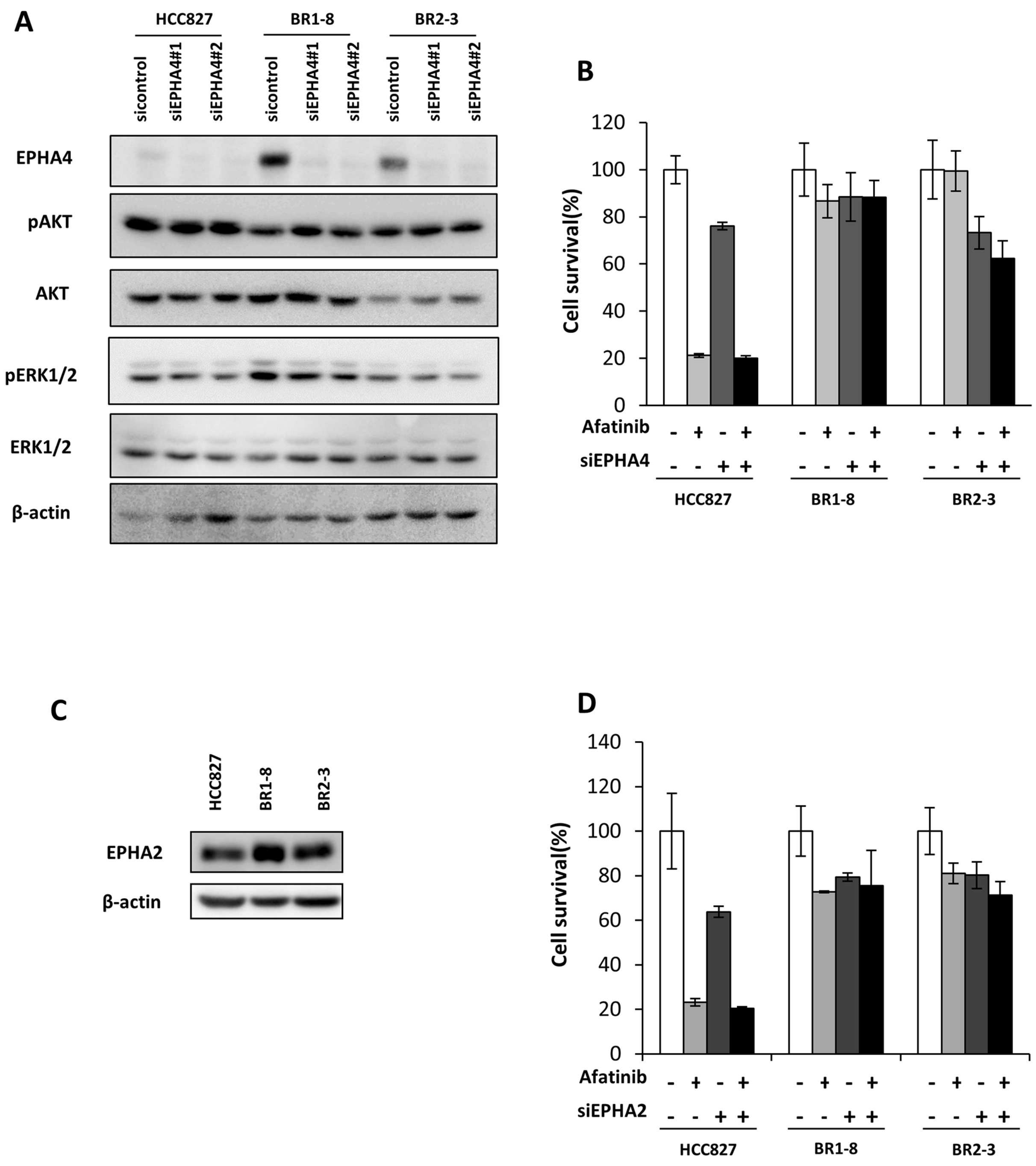


**Supplementary Figure 4: Increased expression of EPHA4 and EPHA2 in drug-resistant sublines.** **(A)** The inhibitory effects of an *EPHA4* siRNA on the phosphorylation of AKT, and ERK in cells transfected for 48 h. **(B)** Effect of an *EPHA4* siRNA on the sensitivities of cells to afatinib. Cells were transfected with *EPHA4* siRNA #1 (5 nmol/L) for 48 h and then treated with afatinib (100 nmol/L) for 72 h. **(C)** The expression of EPHA2, and β-actin served as a loading control. **(D)** Effect of an *EPHA2* siRNA on sensitivity to afatinib. Cells were exposed to a *EPHA2* siRNA (5 nmol/L) for 48 h and then treated with afatinib for 72 h. The data represent the average of triplicate dishes.

**Supplementary Table 1: EGFR gene amplification of HCC827 clones**

| Clone of HCC827 | EGFR/CEP7 ratio ≥ 2 (cells/field) | EGFR/CEP7 ratio < 2 (cells/field) | Not available (cells/field) | Average of EGFR/CEP7 ratio |
|---|---|---|---|---|
| #1 | 80 | 16 | 4 | 5.49 |
| #2 | 82 | 14 | 4 | 5.41 |
| #3 | 86 | 8 | 6 | 6.62 |
| #4 | 90 | 10 | 0 | 4.73 |
| #5 | 68 | 32 | 0 | 3.53 |
| #6 | 86 | 14 | 0 | 5.45 |
| #7 | 78 | 22 | 0 | 5.47 |
| #8 | 86 | 14 | 0 | 4.74 |
| #9 | 82 | 18 | 0 | 4.38 |
| #13 | 14 | 82 | 4 | 1.03 |
